# Arsenic Soil Contamination and Its Effects on 5-Methylcytosine Levels in Onions and Arsenic Distribution and Speciation

**DOI:** 10.3390/toxics11030237

**Published:** 2023-02-28

**Authors:** Marek Popov, Jiří Kudrna, Marie Lhotská, František Hnilička, Barbora Tunklová, Veronika Zemanová, Jan Kubeš, Pavla Vachová, Jana Česká, Lukáš Praus, Karel Štengl, Jiří Krucký

**Affiliations:** 1 Department of Botany and Plant Physiology, Faculty of Agrobiology, Food and Natural Resources, Czech University of Life Sciences, 165 00 Prague, Czech Republic; 2 Department of Agroenvironmental Chemistry and Plant Nutrition, Faculty of Agrobiology, Food and Natural Resources, Czech University of Life Sciences, 165 00 Prague, Czech Republic; 3 Laboratory of Environmental Chemistry, Faculty of Agrobiology, Food and Natural Resources, Czech University of Life Sciences, 165 00 Prague, Czech Republic

**Keywords:** epigenetics, methylation, toxic metals, arsenic accumulation, arsenic species, onion

## Abstract

Arsenic represents a serious health threat in localities with a high arsenic-polluted environment and can easily get into the human food chain through agronomy production in areas affected by arsenic contamination. Onion plants that were grown in controlled conditions in arsenic-contaminated soil (5, 10, and 20 ppm) were harvested 21 days after contamination. Arsenic levels (from 0.43 ± 0.03 µg g^−1^ to 1761.11 ± 101.84 µg g^−1^) in the onion samples were high in the roots and low in the bulbs and leaves, which is probably caused by a reduced ability of the onions to transport arsenic from roots to bulbs and leaves. Arsenic species As(V) and As(III) in As(V)-contaminated soil samples were represented strongly in favor of the As(III) species. This indicates the presence of arsenate reductase. Levels of 5-methylcytosine (5-mC) (from 5.41 ± 0.28% to 21.17 ± 1.33%) in the onion samples were also higher in the roots than in the bulbs and leaves. Microscopic sections of the roots were examined, and the most damage was found in the 10 ppm As variant. Photosynthetic parameters pointed to a significant decrease in photosynthetic apparatus activity and the deterioration of the physiological state of plants as arsenic content increased in the soil.

## 1. Introduction

Arsenic pollution is serious and global environmental problem. More than 200 million people worldwide are affected by high arsenic toxicity and large areas of contaminated soil [1,2,3]. Arsenic is a toxic metalloid with carcinogenic and mutagenic effects and has strong, negative effects on the Human Development Index, public health, and life expectancy [4,5,6,7]. Arsenic accumulated by plants, especially in edible crops, can enter the human food chain and become a possible hazard [8,9,10].

The most common forms of As in the terrestrial environment are arsenate (As(V)) and arsenite (As(III)), whose toxic effects are caused by different mechanisms, such as the inhibition of oxidative phosphorylation by arsenate or the binding of sulfhydryl groups on proteins by arsenite [11,12,13]. Terrestrial plants have evolved protective mechanisms to reduce oxidative stress, usually by transforming inorganic As to less toxic forms, such as reducing arsenate to arsenite by arsenate reductases and complexation with thiolic peptides [12,13,14,15,16,17]. Phytochelatins (PCs), which are synthetized by the transpeptidation of [γ-Glu-Cys-]n-Gly glutathion (GSH) tripeptide monomers (n: 2–11), are most commonly present in PC_2_ and PC_3_. Phytochelatins have an important role in plant defence and protection from arsenic’s toxic effects due to PCs’ high affinity for As(III), not As(V) [18,19,20].

Although many plant species are arsenic resistant and able to survive in soil that is toxic to other plants, the hyperaccumulation of arsenic in plants with no As toxicity symptoms is a rare phenomenon; for example, it occurs in the *Pteridaceae* family [21,22,23]. Plants’ ability to accumulate arsenic is conditioned by how efficiently they translocate arsenic from their roots to their aboveground parts to reduce root damage caused by high arsenic toxicity [8,9,24,25]. Arsenic transporters are key components regarding the accumulation and detoxification of metalloids and their uptake, efflux, and sequestration into intracellular compartments. Arsenate As(V) in plants includes phosphate transporters (PHT1), arsenite As(III) via the aquaporin channels of the Nodulin26-like intrinsic protein subfamily (NIPs), and the plasma membrane intrinsic protein subfamily (PIPs) [8,9,12,13,16,26].

The stress adaptation of plants has epigenetic regulations, including DNA methylation and histone modifications. DNA de-methylation is modification that occurs predominantly on the fifth carbon position of a cytosine ring. It is mediated by activity from methyl group which adds the DNA methyltransferase enzyme family, methyl group removing ten-eleven translocation (TET), and thymine-DNA glycosylase (TDG) enzyme families. Sources of abiotic stress vary in enzyme activity stimulation, lower or increase DNA methylation levels, and alter patterns of gene expression. The demethylation process is coupled with increased gene expression which is in opposition with hypermethylation, inducing gene silencing [27,28,29,30,31,32,33]. Toxic metals can affect methylation levels [34].

This study aimed to explore global methylation levels in different parts of onions that grew in different contaminated soils, their total arsenic accumulation, the representation of arsenic species, and the effect of arsenic contamination on the physiological parameters of plants.

## 2. Materials and Methods

### 2.1. Plant Material

Onions were grown under a 600 W high pressure sodium lamp in a grow box with a 12 h/12 h light/dark cycle at 20 °C for 21 days. Soil (pH_KCl_ 5.6) was contaminated 1 week after the onion plants sprouted using dH_2_O solutions of As(V) with Na_2_HAsO_4_·7H_2_O (Alfa Aesar, Ward Hill, MA, USA) to acquire final concentrations of 5, 10, and 20 ppm of As(V) in the soil. The control sample had no added arsenic for comparison purposes. The samples were collected 21 days after contamination and stored in −80 °C. The samples that were used for the ICP-MS analyses of total arsenic content and the speciation analyses were oven dried (FD 53, Binder, Tuttlingen, Germany) at 40 °C for 72 h to a constant weight.

### 2.2. Analysis of Total Arsenic Content

A 150 mg aliquot of plant biomass was weighed in a 35 mL quartz vessel, then 4 mL of HNO_3_ (Analytika, Prague, Czech Republic) and 2 mL of H_2_O_2_ (Carl Roth, Karlsruhe, Germany) were added. The vessels were capped and heated in a microwave oven (Discover SP-D, CEM Corp., Matthews, NC, USA) at 180 °C for 18 min. The concentration of As in the digested samples that were diluted with Milli-Q water (≥18.2 MΩ cm^−1^; MilliQ system, Millipore SAS, Molsheim, France) was measured using an inductively coupled plasma mass spectrometer (ICP-MS; Agilent 7700x, Agilent Technologies Inc., Santa Clara, CA, USA) that operated in He mode, and an external calibration technique and ^72^Ge were used as the internal standard. For quality assurance of the analytical results, peach leaves were used as a certified reference material (SRM-1547, NIST), and procedural blanks were analysed in three biological replicates.

### 2.3. Arsenic Speciation Analysis

The HPLC system was equipped with an anion exchange column PRP-X100 (150 × 4.6 mm, 10 µm; Hamilton, USA), which was eluted with a gradient with 4 mmol L^−1^ NH_4_NO_3_ (A) and 60 mmol L^−1^ NH_4_NO_3_ (B) at a flow rate of 0.95 mL^−1^ min^−1^. The pH of both mobile phases was adjusted to 8.7 (NH_4_OH), similarly to [35]. The sample injection volume was 20 µL, and the column was heated to 30 °C. The gradients were 70%/30% A/B (0–3.3 min) and 20%/80% A/B (3.5–8.0 min); then, the gradient returned to its initial condition of 70%/30% A/B. Standard solutions at 0.1, 0.5, 2.5, 10, and 50 µg L^−1^ As of each chemical species to be analysed were prepared by dissolution and dilution of NaAsO_2_ (As^III^), Na_2_HAsO_4_ × 7H_2_O (As^V^), and dimethylarsinic acid (DMA), which were all purchased at Fluka (Switzerland), while monomethylarsonate (MMA) was synthesized in-house. All samples were diluted with a mobile phase (50%/50% A/B) to fit into the range of the ICP-MS external calibration. For ICP-MS calibration was used certified reference material ASTASOL-As (1000 ± 2 mg L^−1^), (Analytika, Prague, Czech Republic).

### 2.4. Determination of Gas Exchange Parameters

LCpro+ portable photosynthesis system (ADC BioScientific, Ltd., Hoddesdon, UK) was used to determine the photosynthetic rate (P_n_; µmol CO_2_^−1^ m^−2^ s^−1^), the transpiration rate (E; mmol H_2_O m^−2^ s^−1^), the intercellular CO_2_ concentration (Ci; µmol CO_2_ mol^−1^), and the stomatal conductance (g_s_; mol H_2_O m^−2^ s^−1^) and to calculate instantaneous water-use efficiency (WUE; WUE = P_n_/E). The conditions in the chamber were: 25 °C; an ambient CO_2_ concentration of 550 ± 50 µL L^−1^; an air flow rate of 205 ± 30 µmol s^−1^; and irradiance 650 ± 50 µmol^−1^ m^−2^ s^−1^ of photosynthetically active radiation.

### 2.5. Determination of Pigments

The content of the pigments, including α-chlorophyll, β-chlorophyll, total chlorophyll, and total carotenoids, was measured spectrophotometrically (Evolution 210, Thermo Fisher Scientific Inc., Waltham, MA, USA) in leaf extract of 0.01 g of dry matter (DM) in 1 mL of dimethylformamide and extracted at 24 h and supported with shaking. The centrifuged (Frontiers 5718R, Ohaus, Nanikon, Switzerland) supernatant of the extract was measured at 480, 646.8, and 663.8 nm wavelengths and reduced by 710 nm wavelength values using methods for determinations and calculations of chlorophylls [36] and carotenoids [37].

Chlorophyll a: Chl a; μg mL^−1^: Chl a = 12.0 × A663.8−3.11 × A646.8

Chlorophyll b: Chl b; μg mL^−1^: Chl b = 20.78 × A646.8−4.88 × A663.8

Total chlorophyll: Chlt; μg mL^−1^: Chl a + Chl b = 17.67 × A646.8 + 7.12 × A663.8

Carotenoids: Car; μg mL^−1^: Car = (1000 × A480−1.12 Chl a−34.07 Chl b)/245

### 2.6. Determination of Fluorescence

Leaves were set up in a dark-adapted state by clip-covering for 20 min. The fluorescence of the chlorophyll was excited by a 660 nm solid-state light source with filters that blocked radiation longer than 690 nm. Saturation of the measured photosystem was achieved by using a filtered 35 W halogen lamp (350–690 nm) with a pulse of 15,000 µmol m^−2^ s^−1^ at 0.8 s. The fluorescence of the chlorophyll was measured using a fluorometer OS5p+ (Opti-Sciences Inc., Hudson, NH, USA).

### 2.7. 5-methylcytosine Levels Determination

Samples of onions (roots, bulbs, and leaves) were crushed with a mortar and pestle in liquid nitrogen conditions. Each sample had a wet weight of 100 mg and was used for DNA isolation using a NucleoSpin Plant II isolation kit (Macherey-Nagel GmbH & Co. KG, Dueren, Germany) with miniprep protocol that was recommended by manufacturer using PL1 lysis buffer. Isolated DNA samples were used to determine the global DNA methylation status using a MethylFlash Methylated DNA Quantification Kit (Fluorometric) (Epigentek Group Inc., Farmingdale, NY, USA) and the manufacturer’s attached protocol, and 100 ng of DNA was used for each assay. Fluorescence measurement at 530_EX_/590_EM_ nm was set on the fluorescence microplate reader (Tecan Infinity M200, Tecan Deutschland GmbH, Crailsheim, Germany) using Magellan software.

### 2.8. Microscopy

Transverse microscopic sections were obtained from main cut root, which was approximately 10 mm from the first lateral roots, towards the tip of the root, and observed unstained at 100× and 400× magnification. Four variants, including the control and 5, 10, and 20 ppm of arsenic soil concentrations, were observed using a microscope (Nikon Eclipse 50i with Nikon DS-Fi2 camera, Nikon Corporation, Tokyo, Japan). Microscopic sections of all the variants were monitored based on the following parameters: the rhizodermis with a primary cortex, the endodermis, and the vascular bundle and development of the xylem.

### 2.9. Statistical Analyses

Factorial and one-way ANOVA were used to evaluate differences in the monitored treatments which were defined as the rate of stress by As. After obtaining significant results (*p* < 0.05), multiple comparisons using the Tukey HSD test were applied to identify significant differences between the treatments. These analyses were performed using STATISTICA Version 13 (Statsoft, Tulsa, OK, USA). Relationships between all the variables (Fv/Fm; ci; E; gs; A; chl a; chl b; chl tot; carotenoids; As; 5-mC %) were evaluated via principal component analysis with supplementary variables (stress levels by As) in Canoco 5 [38]. This analysis was appropriate for finding the differences in reactions of the onions in the treatments (control, 5, 10, and 20 ppm).

## 3. Results

### 3.1. Arsenic Accumulation and Speciation in Different Parts of Plants

**Figure 1:**
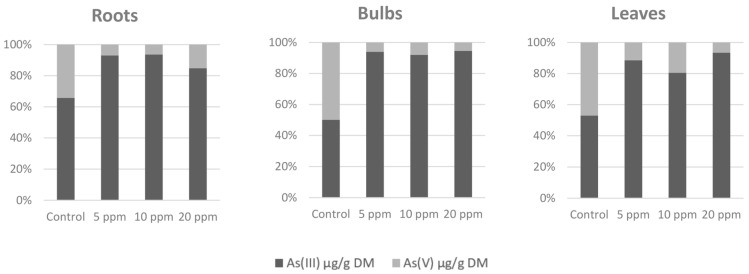
Arsenic species ratio (%).

Arsenic accumulation (Table 1) was most significant in the roots. Almost all arsenic was accumulated in the roots, and for the contaminated variants, the share of arsenic transported to the bulbs and leaves was units of percent only. The content of arsenic in the leaves was slightly higher than in the bulbs where the arsenic content was the lowest. Arsenic species (Figure 1) As(V) and As(III) in the samples from the As(V)-contaminated soil were represented strongly in favor of the As(III) species as more was present in the contaminated variants instead of the control variant. This indicates the presence of arsenate reductase and its increased activity in an arsenic-contaminated environment.

### 3.2. Physiological Responses to Arsenic Contamination

**Figure 2:**
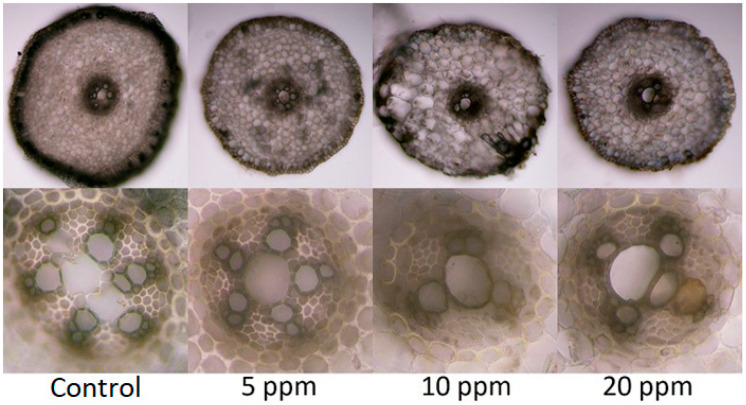
Differences in the arrangement of the rhizodermis with a primary cortex, endodermis, and vascular bundle. Magnification: upper images (100×) and lower images (400×).

When compared with measured parameters of the photosynthetic apparatus condition (Table 2), some differences were found in the gas exchange parameters of individual variants. WUE and Ci increased by 307% and decreased by 29% in the most contaminated variant, respectively, compared with the control variant. Values of E decreased by 28% compared with the control variant. There were no differences found in P_n_ and Fv/Fm, and values of g_s_ were generally very low. Chlorophylls content (Table 2) did not show a clear trend, but the lowest values were found in the 10 ppm variant, which correlates with the microscopy observation results (Figure 2) where the 10 ppm variant was the most affected.

### 3.3. Changes in 5-Methylcytosine Level under Arsenic Exposure

**Figure 3:**
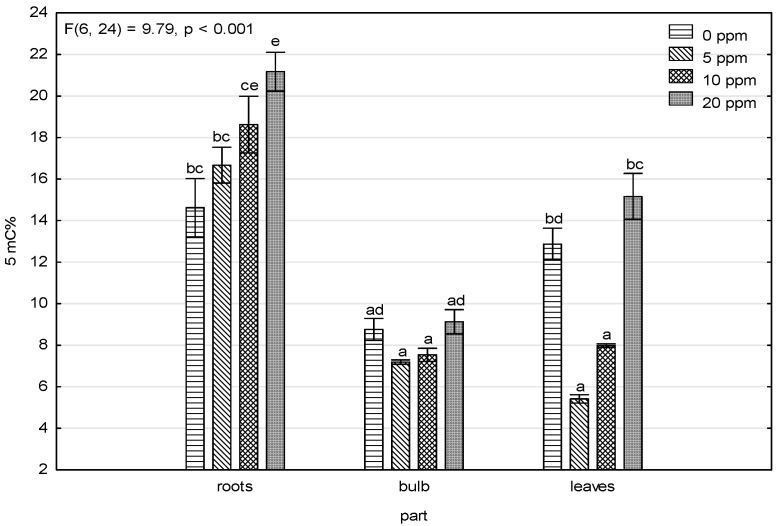
Results of the factorial ANOVA analysis for levels of 5-methylcytosine. The error bars indicate the standard errors, and the bar height is the mean value. Categories on the X-axis show parts of the plant for four treatments (control, 5 ppm, 10 ppm, and 20 ppm). The letters indicate significant differences based on the post-hoc Tukey test, assuming *p* < 0.05.

Figure 3 shows that the 5-methylcytosine levels and arsenic content in the roots of the contaminated variants, where 5-methylcytosine levels had an increasing trend, pointed to DNA hypermethylation caused by high arsenic levels induced by stress. This trend was not shown in the bulbs and leaves, and the amount of accumulated arsenic in the bulbs and leaves (Table 1) was significantly lower. The only similarity was lower methylation levels in the 5 and 10 ppm As(V)-contaminated variants and higher levels in the control and 20 ppm As(V)-contaminated variant, which showed up more in the leaves and less in the bulbs.

### 3.4. Anatomic Changes in Tissues of Plants Exposed to Arsenic

Contamination of the substrate was manifested by certain anatomical changes (Figure 2) in the tissues of the adventitious roots of the onions in all experimental variants compared with the control variant in all monitored parameters. The changes were particularly noticeable in the xylem part of the vascular bundle and the number of xylem poles and in the differentiation of meta and protoxylem. The level of thickening of the outer cell walls of the rhizodermis and the differentiation of the exodermis also differed. The 10 ppm As variant in the substrate reacted most sensitively, and an anomaly appeared in the cortex parenchyma. Regarding the visual assessment of the root system, the shortest and weakest roots were observed in the variant with 20 ppm As in the substrate, which contradicts the greatest anatomical changes shown in the 10 ppm variant. It follows from the evaluation that all three experimental variants responded to the risk factor load, which is evidenced by the observed deviations in all the monitored parameters.

## 4. Discussion

The results were compared with other studies which focused on toxic metal contamination and found a similar range of values [39,40]. The results were compared with studies on the edible parts of onions and showed similarities which confirmed the results of this study. The onions from the control variant had a low arsenic content, which reflected onions from studies monitoring levels of arsenic in uncontaminated areas under a hygienic limit (1 µg g^−1^) [39,40] and was opposite of onions from contaminated areas that found a similar range of values (28.7 µg g^−1^ to 91.6 µg g^−1^) [41,42] in the bulbs and leaves, similar to our contaminated variant samples. These values were significantly lower than values found in similar roots [43] where most of the accumulated arsenic remained.

**Figure 4:**
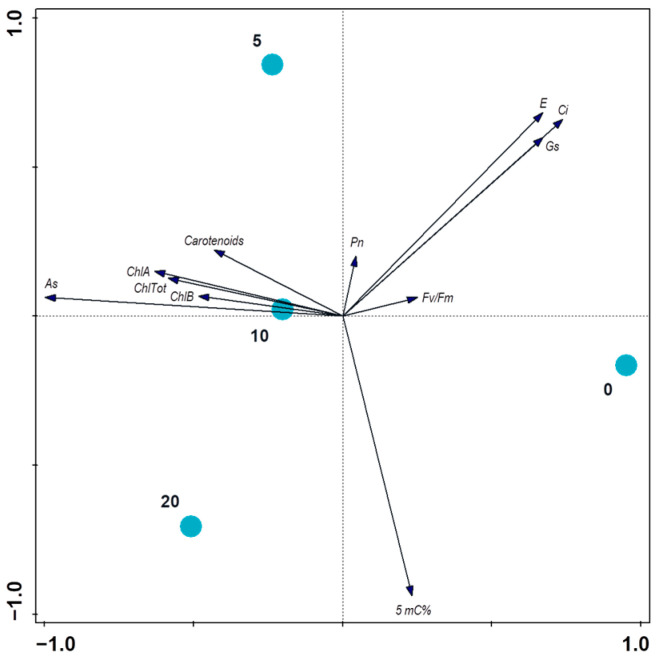
Relationships between the treatments and measured properties of the onions evaluated using PCA (principal component analysis) with supplementary variables. The treatments are displayed using color circles and the arrows are measured variables or properties of the plants. The first two axes explain 97.03% of the total variation in the plants’ properties.

This finding can be interpreted as follows: onions should not be considered an hyperaccumulator plant with an ability to translocate risk elements into aboveground parts of the plants. Even if the values of the accumulated arsenic in the bulbs and leaves are not negligible, they are far from approaching the rates that are typical for arsenic hyperaccumulators (1000 As µg g^−1^ DM) [44]. Most arsenic was found in the roots, which caused the most damage and was clearly visible from observation under a microscope. That has negative effects on the entire plant which are manifested by the following: deteriorated photosynthetic parameters (Table 2); (Figure 4) a decreased transpiration rate (28%) caused by a limited stomatal capability due to arsenic toxicity damage [45]; increased WUE values (307%) caused by arsenic-induced water deficit conditions, which were also observed in toxic metal-stressed plants [46]; and a decreased amount of CO_2_ in the mesophyll cells (29%), pointing to a reduced ability of CO_2_ to fix the arsenic damaged photosynthetic apparatus [47].

The main purpose of the study was to determine whether epigenetic changes occur, and the results of the measurements point to changes in the content of methylated DNA (Figure 3). Plants that are stressed with toxic metals could react differently based on methylate or de-methylate DNA [48,49]. The most visible trend showed that arsenic contamination in soil affects increasing DNA methylation levels in roots where 5-mC content increases as arsenic soil contamination increases. In general, DNA hypermethylation causes lowered gene expression [33]; more specifically, it downregulates most genes connected to the oxidative stress regulation metabolism and main hormone signaling pathways, as shown in [50], with an exception of arsenate reductase. Some plants exposed to arsenate stress have an induced expression and increased activity of arsenate reductase, as shown in [51,52], which transform arsenate As(V) to arsenite As(III). Arsenic species As(V) and As(III) ratios in samples from As(V)-contaminated soil were represented strongly in favor of As(III) species, and there was more in the contaminated variants than the control variant, which indicates an increase in arsenate reductase activity due to the additional arsenic contamination.

The observation of microscopic sections of the roots found that the 10 ppm As(V)-contaminated variant was the most affected and had signs of cytotoxicity, which may be caused by the As(III) that is present in the roots as a major arsenic specie. Other studies that monitored As(III) concentrations and their influence on onion roots showed a similar trend in monitored plant responses [53,54] as variants with lower (5 and 20 mg L^−1^) and higher (3 and 30 mg L^−1^) As(III) contamination showed a smaller cytotoxicity response than variants between them (10 mg L^−1^) in both cases during 1–12 h observations. However, this can be exhausting for the plants over a longer period, such as the 21 days used in this experiment, and can make them more sensitive to the other harmful effects of arsenic.

## 5. Conclusions

An upward trend was found in DNA methylation levels which increased as values of accumulated arsenic increased. This finding mainly occurred in the roots, which also had the highest ability to accumulate arsenic and was different than the arsenic content values measured in the bulbs and leaves. Arsenic species As(V) and As(III) ratios in samples from As(V)-contaminated soil were represented strongly in favor of As(III) species, which highlights arsenate reductase activity. Root tissue damage was also found. Root development was disrupted by the presence of arsenic, which is similar to the photosynthetic apparatus as its activity was significantly attenuated because of arsenic exposure.

## Figures and Tables

**Table 1:** Arsenic content in different parts and variants (As µg g^−1^ DM).

ppm As(V)	Roots	Bulbs	Leaves
**Control**	2.67 ± 0.11^a^	0.60 ± 0.01^b^	0.43 ± 0.03^a^
**5**	1488.29 ± 23.31^b^	10.39 ± 0.22^c^	28.76 ± 2.06^b^
**10**	1249.82 ± 41.75^c^	26.94 ± 0.59^a^	23.44 ± 0.13^c^
**20**	1761.11 ± 101.84^d^	28.06 ± 1.29^a^	44.74 ± 1.27^d^
**P**	<0.001	<0.001	<0.001

The letters indicate significant differences based on the post-hoc Tukey test, assuming *p* < 0.05.

**Table 2:** Chlorophyll fluorescence, gas exchange parameters, and pigment content.

Parameter	Control	5 ppm As(V)	10 ppm As(V)	20 ppm As(V)	*p*
F_v_/F_m_	0.77 ± 0.00	0.75 ± 0.02	0.78 ± 0.02	0.75 ± 0.02	0.446
Ci	536.67 ± 50.63 ^b^	444.33 ± 26.52 ^b^	279.67 ± 17.57 ^a^	154.67 ± 10.27 ^a^	<0.001
E	1.77 ± 0.11 ^b^	1.73 ± 0.05 ^b^	0.75 ± 0.03 ^a^	0.50 ± 0.02 ^a^	<0.001
g_s_	0.07 ± 0.00 *^b^	0.06 ± 0.00 *^b^	0.02 ± 0.00 *^a^	0.02 ± 0.00 *^a^	<0.001
P_n_	6.77 ± 1.88	7.31 ± 0.69	5.54 ± 0.54	6.03 ± 0.60	0.677
WUE	3.99 ± 1.39 ^a^	4.25 ± 0.52 ^a^	7.37 ± 0.56 ^ab^	12.24 ± 1.58 ^b^	0.003
Chl *a*	0.83 ± 0.02 ^b^	1.45 ± 0.04 ^d^	0.80 ± 0.02 ^a^	1.42 ± 0.05 ^c^	<0.001
Chl *b*	0.29 ± 0.01 ^b^	0.49 ± 0.02 ^c^	0.22 ± 0.01 ^a^	0.50 ± 0.02 ^d^	<0.001
Chl_t_	1.12 ± 0.03 ^b^	1.94 ± 0.05 ^d^	1.02 ± 0.02 ^a^	1.92 ± 0.06 ^c^	<0.001
Car	0.17 ± 0.01 ^b^	0.26 ± 0.02 ^d^	0.14 ± 0.01 ^a^	0.24 ± 0.01 ^c^	<0.001

* SD < 0.005. F_v_/Fm: maximum quantum yield of the photosystem; Ci: intercellular CO_2_ (µmol CO_2_ mol^−1^); E: transpiration rate (mmol CO_2_ m^−2^ s^−1^); g_s_: stomatal conductance (mol H_2_O m^−2^ s^−1^), P_n_: net photosynthetic rate (µmol CO_2_ m^−2^ s^−1^); WUE: water use efficiency (P_n_/E) (µmol CO_2_ mmol^−1^ H_2_O); Chl *a*: chlorophyll *a* (mg g^−1^ DM); Chl *b*: chlorophyll *b* (mg g^−1^ DM); Chl_t_: total chlorophyll (mg g^−1^ DM); and Car: total carotenoids (mg g^−1^ DM). The letters indicate significant differences based on the post-hoc Tukey test, assuming *p* < 0.05.

## Data Availability

The data presented in this study are available in this article.
